# Predicting the recurrence of usual-type cervical adenocarcinoma using a nomogram based on clinical and pathological factors: a retrospective observational study

**DOI:** 10.3389/fonc.2024.1320265

**Published:** 2024-02-07

**Authors:** Yuting Liu, Ningning Zhang, Qing Yang

**Affiliations:** Department of Obstetrics and Gynecology, Shengjing Hospital of China Medical University, Shenyang, China

**Keywords:** usual-type cervical adenocarcinoma, nomogram, retrospective, observational, recurrent cervical cancer

## Abstract

**Background:**

Usual-type cervical adenocarcinoma is the most frequent type of adenocarcinoma, and its prevalence is increasing worldwide. Tumor recurrence is the leading cause of mortality; therefore, recognizing the risk factors for cervical cancer recurrence and providing effective therapy for recurrent cervical cancer are critical steps in increasing patient survival rates. This study aimed to retrospectively analyze the clinicopathological data of patients with usual-type cervical adenocarcinoma by combining the diagnosis and treatment records after the initial treatment and recurrence.

**Methods:**

We retrospectively analyzed patients diagnosed with usual-type cervical adenocarcinoma who underwent radical hysterectomy and pelvic lymph node dissection at Shengjing Hospital of China Medical University between June 2013 and June 2022. We constructed a nomogram-based postoperative recurrence prediction model, internally evaluated its efficacy, and performed internal validation.

**Results:**

This study included 395 participants, including 87 individuals with recurrence. At a 7:3 ratio, the 395 patients were divided into two groups: a training set (n = 276) and a validation set (n = 119). The training set was subjected to univariate analysis, and the risk variables for recurrence included smoking, ovarian metastasis, International Federation of Gynaecology and Obstetrics (FIGO) staging, lymphovascular space invasion, perineural invasion, depth of muscular invasion, tumor size, lymph node metastasis, and postoperative HPV infection months. The aforementioned components were analyzed using logistic regression analysis, and the results showed that the postoperative HPV infection month, tumor size, perineural invasion, and FIGO stage were independent risk factors for postoperative recurrence (p<0.05). The aforementioned model was represented as a nomogram. The training and validation set consistency indices, calculated using the bootstrap method of internal validation, were 0.88 and 0.86, respectively. The model constructed in this study predicted the postoperative recurrence of usual-type cervical cancer, as indicated by the receiver operating characteristic curve. The model demonstrated good performance, as evidenced by the area under the curve, sensitivity, and specificity values of 0.90, 0.859, and 0.844, respectively.

**Conclusion:**

Based on the FIGO staging, peripheral nerve invasion, tumor size, and months of postoperative HPV infection, the predictive model and nomogram for postoperative recurrence of usual-type cervical adenocarcinoma are precise and effective. More extensive stratified evaluations of the risk of cervical adenocarcinoma recurrence are still required, as is a thorough assessment of postoperative recurrence in the future.

## 1 Introduction

According to the International Agency for Research on Cancer, cervical cancer is the fourth leading cause of cancer-related deaths in women, with 604,000 new cases and 342,000 deaths reported worldwide in 2020 (1). Most patients with cervical cancer have a fair prognosis after standard treatment; however, some patients have a poor prognosis owing to recurrence or specific pathological types. Squamous carcinoma (75%) and adenocarcinoma (20%) are the most common histological types of cervical cancer.

Cervical adenocarcinoma is difficult to diagnose using cervical cytology because the lesions are hidden, the heterogeneous changes in the nucleus of exfoliated cells are not as prominent as in squamous carcinoma, and some adenocarcinoma results are negative in human papillomavirus (HPV) screening. In recent years, the incidence of squamous carcinoma has decreased but that of adenocarcinoma and other types of cervical cancer has gradually increased, with the usual type being the most prevalent (2).

Despite standardized initial therapy, 10–50% of patients with cervical cancer experience recurrence (3, 4), and the long-term survival rate for patients with recurrence is only 10–20% (5). Tumor recurrence is the leading cause of death in patients with cervical cancer. Predicting the risk factors for cervical cancer recurrence; monitoring management, such as regular re-examination of HPV mRNA; and effectively treating cervical cancer recurrence are critical for improving patient survival. The 5-year survival rates of patients with cervical cancer recurrence range from 15 to 50% (6, 7). The risk factors for usual-type cervical adenocarcinoma recurrence after surgery are not currently the subject of any research. This study aimed to retrospectively analyze the clinicopathological data of patients with usual-type cervical adenocarcinoma by combining the diagnosis and treatment records after the initial treatment and recurrence. Statistical methods were used to explore the risk factors for the postoperative recurrence of usual-type cervical adenocarcinoma. Additionally, a model for postoperative recurrence and prognosis after recurrence was constructed. This model can provide a basis for the long-term management of postoperative patients with usual-type cervical adenocarcinoma.

## 2 Materials and methods

### 2.1 Study design

We identified the independent risk factors for eventual recurrence by retrospectively evaluating data of patients with usual-type cervical adenocarcinoma diagnosed and treated at Shengjing Hospital, monitoring their postoperative recurrence, and dividing them into the recurrence and non-recurrence groups based on follow-up outcomes.

### 2.2 Study participants

This study included patients who underwent radical hysterectomy and pelvic lymph node dissection at Shengjing Hospital of China Medical University between June 2013 and June 2022 and were diagnosed with usual-type cervical adenocarcinoma by pathologists.

The inclusion criteria were as follows: (1) pathological diagnosis of usual-type cervical adenocarcinoma at our hospital and confirmation by two pathologists for examination, (2) treatment at our hospital with complete case data, (3) surgical standardization of treatment according to the National Comprehensive Cancer Network (NCCN) guidelines and staging (Ia2 and above) performed according to the 2018 International Federation of Gynaecology and Obstetrics (FIGO) staging, and (4) postoperative recurrence defined as the reappearance of a tumor lesion of the same histological type after 6 months of surgical treatment to achieve clinical recovery, as established in this study by histology or imaging (computed tomography [CT]/positron emission tomography-CT).

The exclusion criteria were as follows: (1) loss of visits, (2) coexistence with other malignant tumors, and (3) coexistence with other serious medical or surgical diseases.

### 2.3 Methods

We mainly conducted follow-ups via telephone and gathered treatment data from the hospital’s information system. From Shengjing Hospital’s information system, 442 individuals with usual-type cervical cancer were selected. After excluding 14 patients whose clinical records were incomplete, 428 patients were contacted for follow-up. Of these, three declined to be followed up, one died in an accident, 29 were lost to follow-up, and 395 were eventually included, yielding an 89% follow-up rate. The final study participants were randomly divided into a training set and a validation set at a 7:3 ratio. Subsequently, the two groups were compared. The training set was subjected to univariate analysis, and significant items were included in a multifactor analysis for additional examination. The validation set was subjected to internal validation.

In this study, the Virus Research Laboratory of Shengjing Hospital detected HPV DNA using hybrid capture technology on cervical exfoliated cytology. Researchers conducted testing and reported the results within three months before surgery and three to six months after surgery, in accordance with our unit’s clinical testing guidelines. Currently, 18 high-risk HPV subtypes (HPV16, 18, 26, 31, 35, 39, 45, 51, 52, 53, 56, 58, 59, 66, 68, 73, and 82) and 11 low-risk HPV subtypes (HPV6, 11, 40, 42, 44, 54, 61, 70, 72, and 81) can be identified.

The annual NCCN guidelines served as the foundation for adjuvant therapy, with particular attention to the high-risk characteristics of lymph node positivity, positive resection margins, and parauterine invasion. If any one of these conditions is met, postoperative concurrent chemotherapy with cisplatin and further external pelvic irradiation is administered. Adenocarcinoma, tumor diameter >3 cm, lymphovascular space invasion (LVSI), and tumor invasion of the outer one-third of the cervical stroma were medium-risk factors. Two of these criteria were met. Therefore, concomitant chemotherapy and external irradiation were administered.

### 2.4 Data collection

The patients who met the inclusion criteria were divided into two groups: those who experienced recurrence and those who did not. Data were retrospectively analyzed to gather demographic information, pathological tumor characteristics, treatment data, and survival rates.

The collected data included the following:

(1) Demographic data, including age, menopause status, body mass index(BMI), smoking status, gravidity, and parity.
(2) Treatment information, including surgical procedures, ovary retention, postoperative adjuvant therapy, targeted therapy, HPV infection, and postoperative HPV persistent infection months (from the day of surgery to the postoperative review of vaginal edge HPV-negative time).
(3) Tumor pathology features, including FIGO stage, degree of differentiation, LVSI, nerve invasion, parametrial invasion, vaginal margin, myometrial invasion, and tumor size.
(4) Survival outcomes, with patients who experienced recurrence after surgery, recording both the type and diagnosis of tumor recurrence.

### 2.5 Statistical analyses

To determine the optimal cutoff value for continuous variables (age, gravidity, parity, and postoperative persistent HPV infection duration), the X-tile software was used. To screen out independent risk variables for recurrence of usual-type cervical cancer, the Statistical Package for the Social Sciences version 26.0 software was used for data analysis. The χ^2^ test was used for univariate analysis, and logistic stepwise backward regression analysis was performed for multivariate analysis. The R.4.3.0 software was used to construct a nomogram and test for predicting the probability of recurrence of usual-type cervical cancer following surgery, as well as for conducting a goodness-of-fit test. The internal validation method was used to recalculate the consistency index (C-index) using bootstrap self-sampling (1000 times) to demonstrate the model’s repeatability.

### 2.6 Ethical consideration

This study was approved by the hospital’s ethics committee (Ethics No. 2023PS890K), and the participants provided informed consent.

## 3 Results

### 3.1 Patients’ basic information

The best age cutoff value was 35 years, the best gravidity cutoff value was 2, the best parity cutoff value was 2, and the number of months of persistent HPV infection after surgery was 9 months. A total of 395 patients were included in this study based on the inclusion and exclusion criteria, with 40 (10.1%) patients aged <35 years and 355 (89.9%) aged >35 years. There were 156 postmenopausal (39.5%) and 239 premenopausal (60.5%) women. 330 (83.5%) women had two or more pregnancies, while 65 (16.5%) women had fewer than two pregnancies. In total, 278 (70.4%)individuals had parity <2 times, whereas 117 (29.6%) individualshad parity ≥2 times. In total, 373 patients were nonsmokers (94.4%) and 22 (5.6%) were smokers. A total of 57 (14.4%) patients underwent laparoscopic surgery, and 338 (85.6%) patients underwent transabdominal surgery.290 patients had cervical exfoliated cell HPvDNA screening performed as part of preoperative HPV testing; 219 (75.51%) had positive results and 71 (24.48%) had negative results. Of the individuals in the recurrence group, 21 individuals, or 36.84% (21/57), tested negative for HPV. Of the non-recurrent group, 50 individuals (21.46%, or 50/233) tested negative for HPV. Recurrence rates were 29.58% (21/71) for negative patients and 16.44% (36/219) for the HPV positive group, respectively. This difference in recurrence rates was statistically significant (p=0.0157). In accordance with the standard, 308 patients did not experience recurrence, and 87 patients experienced recurrence (**Table 1**). Follow-up was allowed for a maximum of 123 (mean, 53) months. In this study, the recurrence rate was 22.0% (87/395) (**Table 1**).

**Table 1:** Demographic characteristics of patients with usual-type cervical adenocarcinoma.

Variable	recurrence group (n = 87)	non-recurrence group (n = 308)	Total (n = 395)	Statistic	P
age, n (%)				χ²=6.206	0.013
>35	72 (82.76)	283 (91.88)	355 (89.87)		
≤35	15 (17.24)	25 (8.12)	40 (10.13)		
gravity, n (%)				χ²=1.998	0.158
<2	10 (11.49)	55 (17.86)	65 (16.46)		
≥2	77 (88.51)	253 (82.14)	330 (83.54)		
parity, n (%)				χ²=0.220	0.638
<2	63 (72.41)	215 (69.81)	278(70.38)		
≥2	24 (27.59)	93 (30.19)	117 (29.62)		
BMI^1^, n (%)				χ²=0.229	0.632
≥28	10 (11.49)	30 (9.74)	40 (10.13)		
<28	77 (88.51)	278 (90.26)	355 (89.87)		
menopause, n (%)				χ²=0.430	0.512
no	50 (57.47)	189 (61.36)	239 (60.51)		
yes	37 (42.53)	119 (38.64)	156 (39.49)		
smoke, n (%)				χ²=8.961	0.003
no	76 (87.36)	297 (96.43)	373 (94.43)		
yes	11 (12.64)	11 (3.57)	22 (5.57)		
Types of HPV^2^, n (%)				χ²=16.319	0.022
16+	11 (19.30)	78 (33.48)	89 (30.69)		
16+18+	1 (1.75)	10 (4.29)	11 (3.79)		
16+18+and other	3 (5.26)	2 (0.86)	5 (1.72)		
16+and other	1 (1.75)	8 (3.43)	9 (3.10)		
18+	12 (21.05)	63 (27.04)	75 (25.86)		
18+and other	2 (3.51)	12 (5.15)	14 (4.83)		
other+	6 (10.53)	10 (4.29)	16 (5.52)		
negative	21 (36.84)	50 (21.46)	71 (24.48)		
Not done	30 (–)	75 (–)	105 (–)		
surgical methods, n (%)				χ²=1.508	0.219
transabdominal	78 (89.66)	260 (84.42)	338 (85.57)		
laparoscopic	9 (10.34)	48 (15.58)	57 (14.43)		
Ovary, n (%)				χ²=0.318	0.573
resection	80 (91.95)	277 (89.94)	357 (90.38)		
retain	7 (8.05)	31 (10.06)	38 (9.62)		
Ovarian metastasis, n (%)				χ²=24.128	<.001
no	75 (86.21)	304 (98.70)	379 (95.95)		
yes	12 (13.79)	4 (1.30)	16 (4.05)		
Degree of differentiation, n (%)				χ²=4.882	0.087
low	35 (40.23)	87 (28.25)	122 (30.89)		
median	17 (19.54)	82 (26.62)	99 (25.06)		
high	35 (40.23)	139 (45.13)	174 (44.05)		
FIGO, n (%)				-	<.001
I	21 (24.14)	231 (75.00)	252 (64.8)		
II	15 (17.24)	46 (14.94)	61 (15.44)		
III	48 (55.17)	31 (10.06)	79 (20)		
IV	3 (3.45)	0 (0.00)	3 (0.76)		
LVSI^3^, n (%)				-	0.517
no	72 (82.76)	268 (87.01)	340 (86.08)		
yes	15 (17.24)	40 (12.99)	55 (13.92)		
Perineural invasion, n (%)				χ²=10.385	0.001
no	77 (88.51)	300 (97.40)	377 (95.44)		
yes	10 (11.49)	8 (2.60)	18 (4.56)		
The depth of myometrial invasion, n (%)				χ²=22.261	<.001
<1/2	22 (25.29)	166 (53.90)	188 (47.59)		
≥1/2	65 (74.71)	142 (46.10)	207 (52.41)		
Tumor size, n (%)				χ²=21.587	<.001
<4cm	50 (57.47)	251 (81.49)	301 (76.2)		
≥4cm	37 (42.53)	57 (18.51)	94 (23.8)		
Lymph node metastasis, n (%)				χ²=89.871	<.001
no	38 (43.68)	277 (89.94)	315 (79.75)		
yes	49 (56.32)	31 (10.06)	80 (20.25)		
Parametrial involved, n (%)				-	0.048
no	85 (97.70)	308 (100.00)	393 (99.49)		
yes	2 (2.30)	0 (0.00)	2 (0.51)		
Vaginal margin, n (%)				χ²=1.369	0.242
no	84 (96.55)	305 (99.03)	389 (98.48)		
yes	3 (3.45)	3 (0.97)	6 (1.52)		
adjuvant treatment, n (%)				-	0.001
C^4^	11 (12.64)	20 (6.49)	31 (7.85)		
C, R^5^	0 (0.00)	4 (1.30)	4 (1.01)		
CCRT^6^	16 (18.39)	46 (14.94)	62 (15.7)		
R^7^	10 (11.49)	43 (13.96)	53 (13.42)		
R,C^8^	9 (10.34)	4 (1.30)	13 (3.29)		
NO^9^	41 (47.13)	191 (62.01)	232 (58.73)		
targeted therapy, n (%)				χ²=0.405	0.525
no	84 (96.55)	303 (98.38)	387 (97.97)		
yes	3 (3.45)	5 (1.62)	8 (2.03)		
HPV persistent infection month, (%)				χ²=67.219	<.001
<9	63 (72.41)	303 (98.38)	366 (92.66)		
≥9	24 (27.59)	5 (1.62)	29 (7.34)		

1: body mass index; 2: preoperative human papillomavirus infection type; 3: lymphovascular space invasion; 4: chemotherapy; 5: chemotherapy then radiotherapy; 6: concurrent chemoradio-therapy; 7: radiotherapy; 8: radiotherapy then chemotherapy; 9: not done.

### 3.2 Logistic regression analysis on postoperative recurrence of usual-type cervical adeno-carcinoma

**Figure 1:**
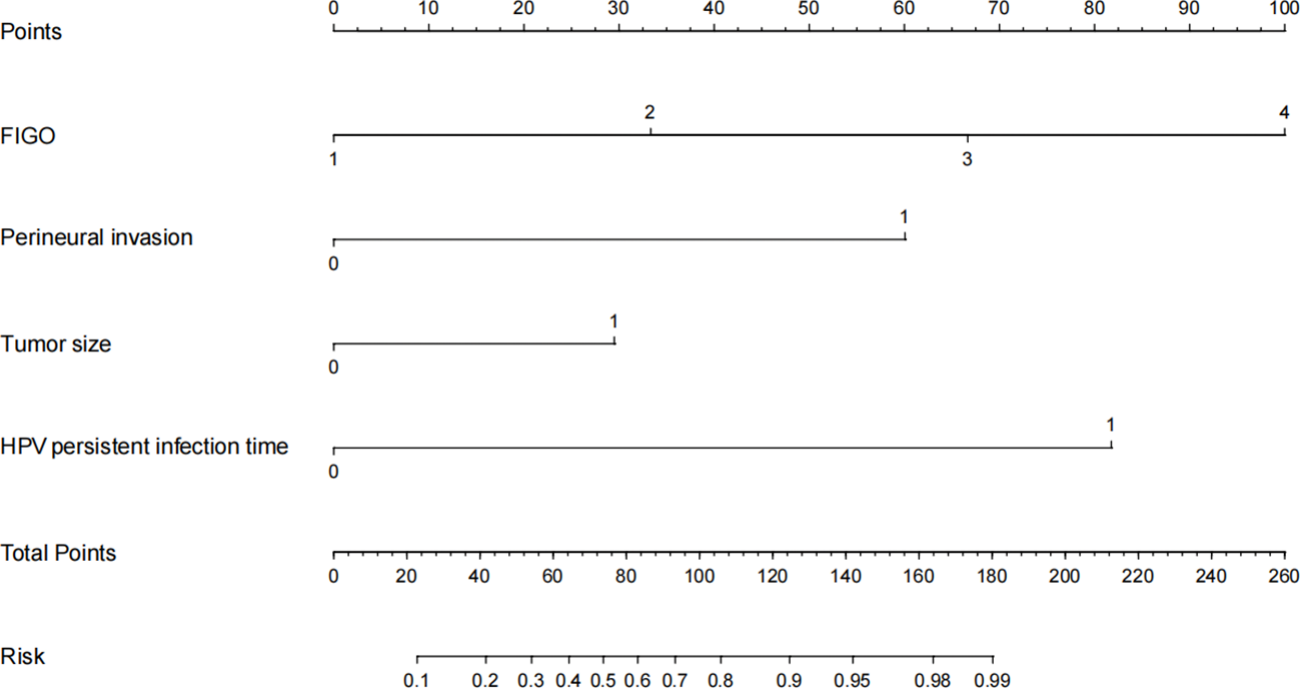
A nomogram for predicting postoperative recurrence of usual-type cervical adenocarcinoma.

A total of 395 patients were randomly divided at a 7:3 ratio into two groups: a training set (n = 276) and a validation set (n = 119). Subsequently, the two groups were compared. There were no significant differences between the two groups in any of the variables (p>0.05), except for age (p<0.05). Univariate analysis was performed on the training set to identify the risk factors for postoperative recurrence, including smoking, ovarian metastasis, FIGO staging, LVSI, perineural invasion, tumor size, lymph node metastasis, month following HPV infection, and depth of myometrial invasion. After applying logistic stepwise regression analysis to the aforementioned data, the following factors were considered the independent risk factors for postoperative recurrence of d usual-type cervical adenocarcinoma (p<0.05): tumor size, perineural invasion, FIGO staging, and month of postoperative HPV infection (**Table 2**). The nomogram for the visualization of the aforementioned model is presented in **Figure 1** and **Table 3**. Furthermore, 1000 internal samples were drawn from the training and validation sets using the bootstrap method of internal validation; this produced C-indices of 0.88 and 0.86, respectively. There was no collinearity interference issue across the variables according to the prediction model constructed in this study.

**Table 2:** Univariate and logistic multivariate regression analysis of the influencing factors of post-operative recurrence of usual-type cervical adenocarcinoma.

Variables	univariate		P	multivariate		P
	Beta	OR (95%CI)		Beta	OR (95%CI)	
Smoke						
no		1.00 (Reference)			1.00 (Reference)	
yes	1.61	5.02 (1.67 - 15.07)	0.004	1.49	4.44 (0.96 - 20.53)	0.056
Ovarian metastasis						
no		1.00 (Reference)			1.00 (Reference)	
yes	2.46	11.67 (3.05 - 44.57)	<.001	1.61	4.98 (0.89 - 27.86)	0.067
FIGO						
I		1.00 (Reference)			1.00 (Reference)	
II	1.30	3.68 (1.59 - 8.50)	0.002	0.99	2.69 (0.95 - 7.61)	0.062
III	2.89	18.00 (8.31 - 38.99)	<.001	2.72	15.18 (5.81 - 39.62)	<.001
IV	17.88	58297855.21 (0.00 - Inf)	0.983	18.29	87356396.05 (0.00 - Inf)	0.989
LVSI^1^						
no		1.00 (Reference)			1.00 (Reference)	
yes	0.76	2.15 (1.04 - 4.44)	0.039	-0.92	0.40 (0.11 - 1.40)	0.151
Perineural invasion						
no		1.00 (Reference)			1.00 (Reference)	
yes	2.87	17.58 (3.69 - 83.76)	<.001	3.10	22.31 (2.77 - 179.70)	0.004
The depth of myometrial invasion						
<1/2		1.00 (Reference)			1.00 (Reference)	
≥1/2	1.46	4.29 (2.26 - 8.14)	<.001	0.83	2.09 (3.75 - 4.70)	0.143
Tumor size						
<4cm		1.00 (Reference)			1.00 (Reference)	
≥4cm	1.34	3.83 (2.05 - 7.15)	<.001	1.41	4.09 (1.73 - 9.70)	0.001
Lymph node metastasis						
no		1.00 (Reference)			1.00 (Reference)	
yes	2.48	11.92 (5.97 - 23.78)	<.001	1.21	3.05 (4.75 - 6.77)	0.243
HPV persistent infection month						
<9		1.00 (Reference)			1.00 (Reference)	
≥9	3.25	25.87 (7.28 - 91.95)	<.001	3.67	39.24 (9.05 - 170.19)	<.001

1: lymphovascular space invasion.

**Table 3:** Assignment description of the nomogram.

Variables	Description of valuation
FIGO	1: I 2: II 3: III 4: IV
Perineural invasion	0: no 1: yes
Tumor size	0: <4cm 1: ≥4cm
Postoperative HPV infection time	0:<no><9</no> months 1: ≥9 months

### 3.3 Model evaluation

**Figure 2:**
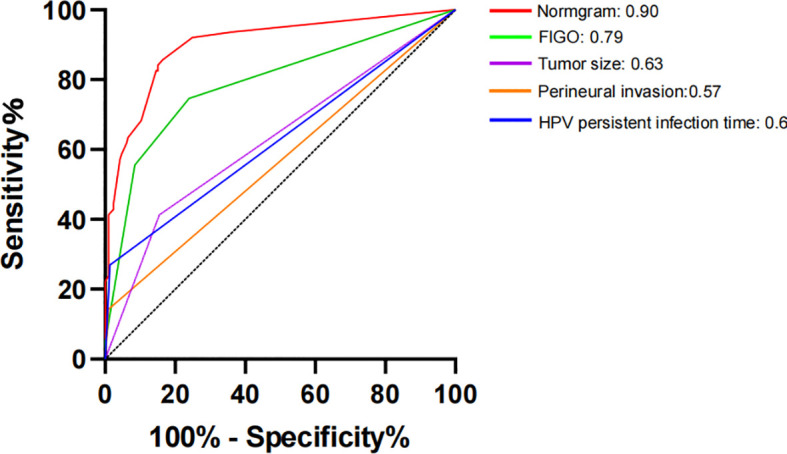
Receiver operating characteristic (ROC) curve of the training set.

**Figure 3:**
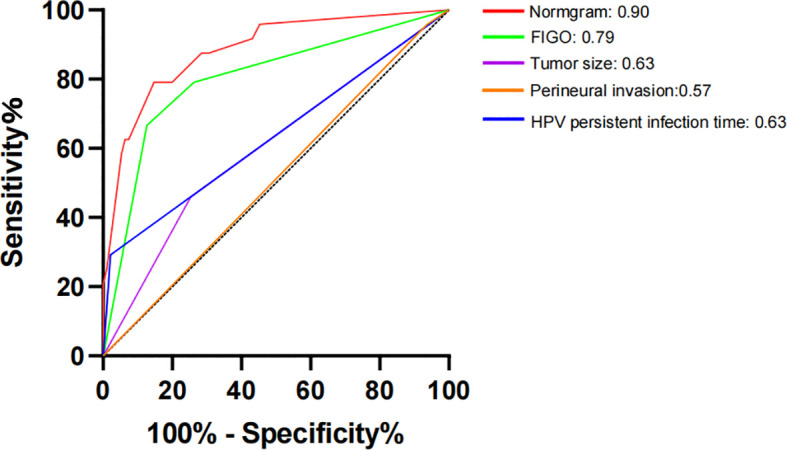
Receiver operating characteristic (ROC) curve of the validation set.

**Figure 4:**
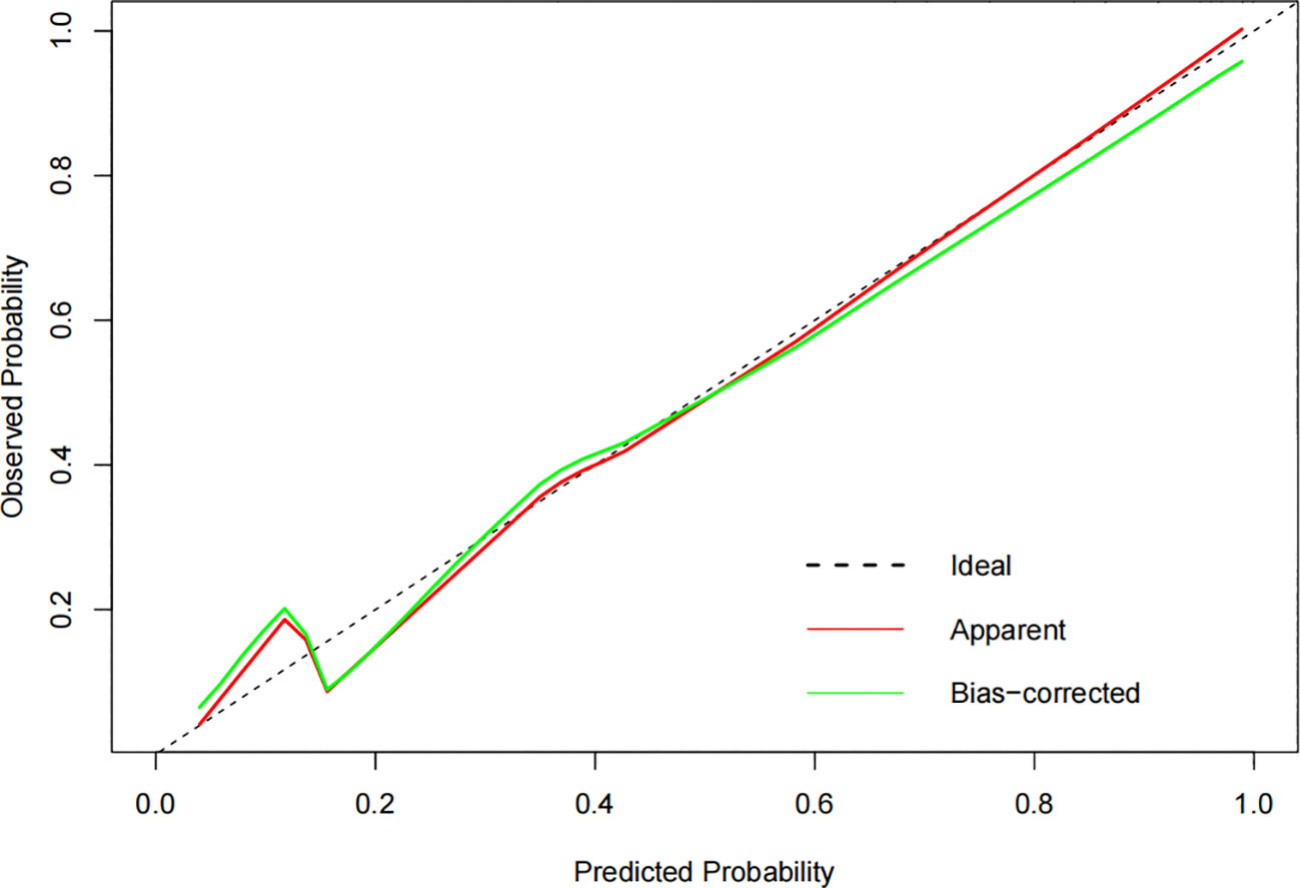
The calibration curves of the training set.

**Figure 5:**
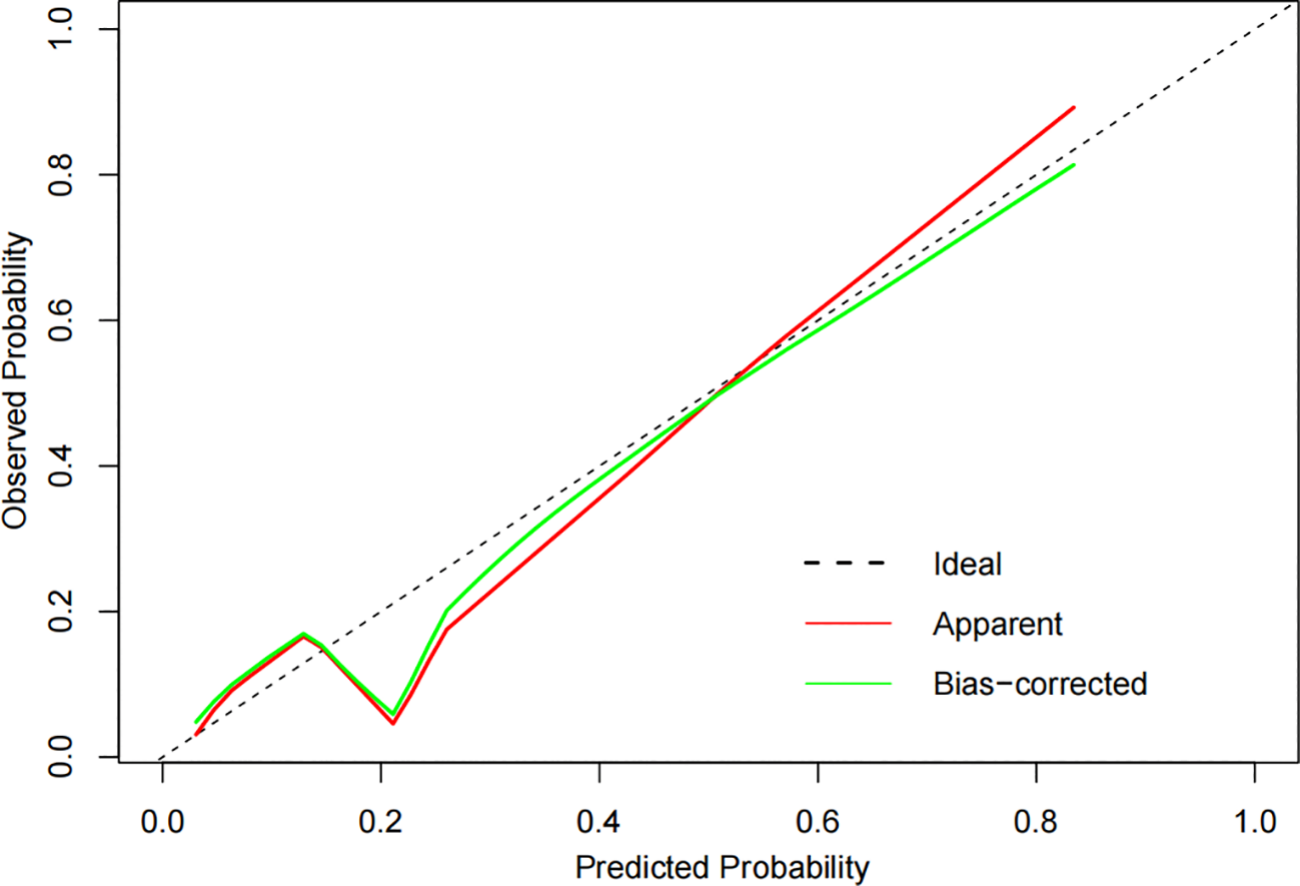
The calibration curves of the validation set.

**Figure 6:**
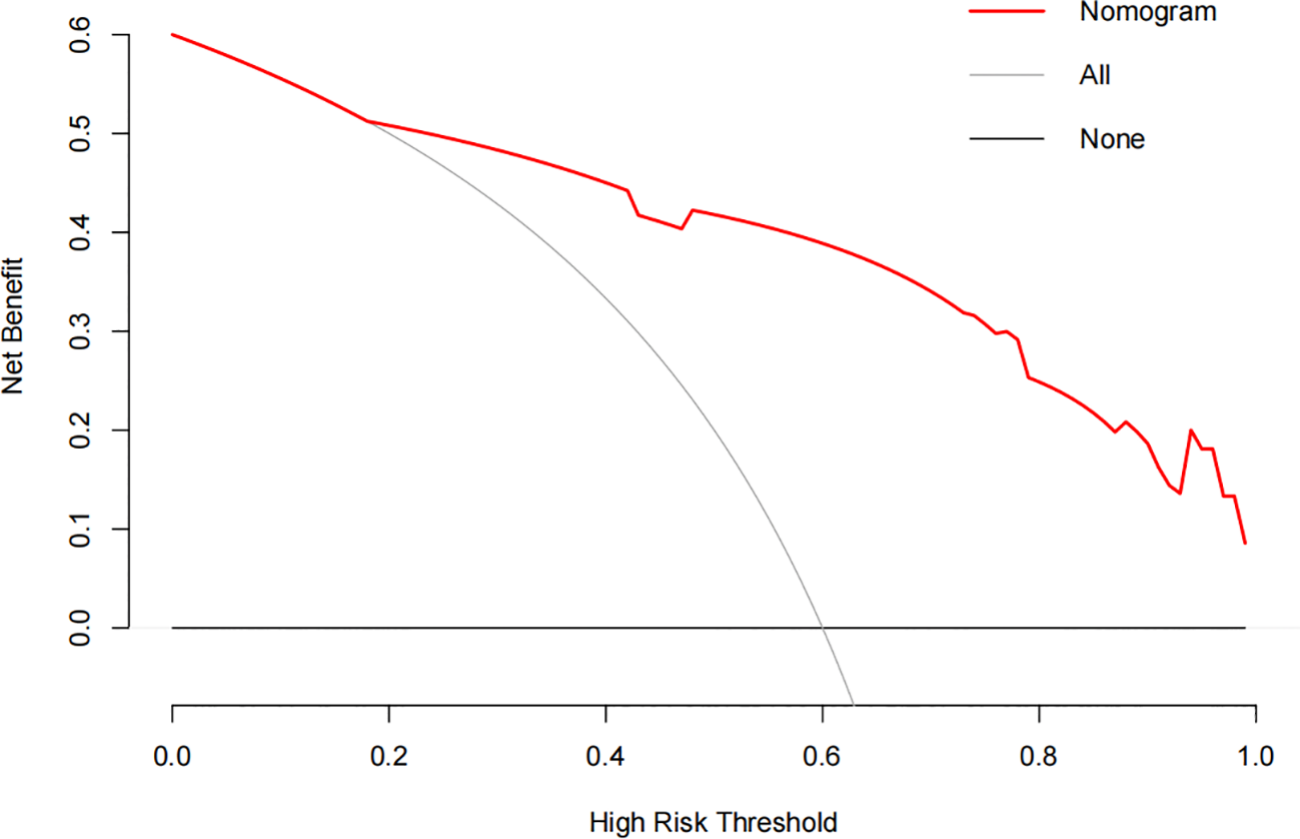
The clinical decision curve analysis of the training set.

**Figure 7:**
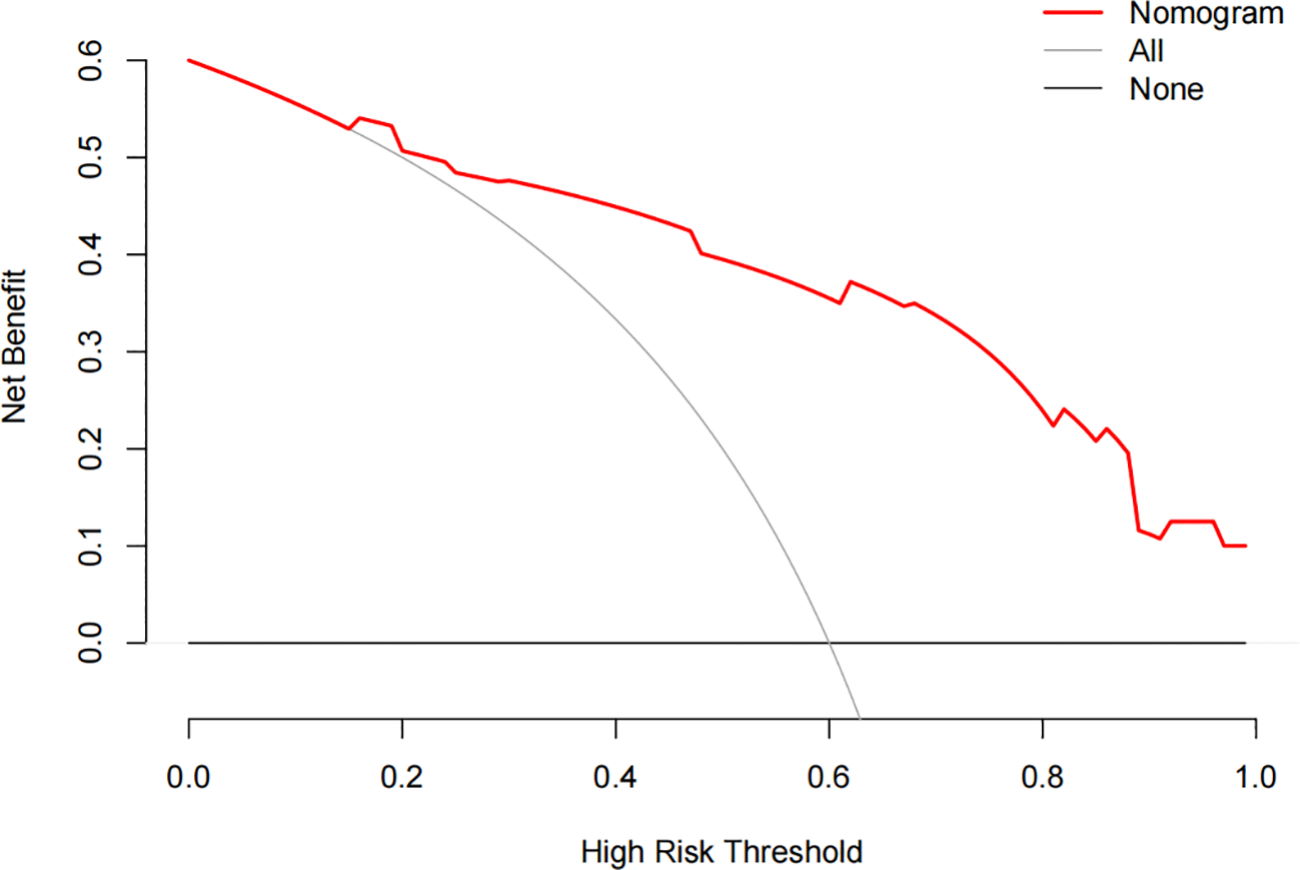
The clinical decision curve analysis of the validation set.

The constructed model had a predictive effect on the postoperative recurrence of usual-type cervical adenocarcinoma according to the receiver operating characteristic (ROC) curve (**Figures 2**, **3**). With an area under the curve (AUC) value of 0.90, the predictive model had good clinical practical value and high discrimination. The model in the validation set also showed good discrimination. The training group’s model performed well, as evidenced by the highest Jordan index of 0.608 and sensitivity, specificity, and accuracy of 0.794, 0.859, and 0.844, respectively. There was no significant difference in the model’s AUC value prediction between the training and validation sets, suggesting that the column chart prediction model had a high degree of repeatability. The calibration curves of the training and validation set prediction models (**Figures 4**, **5**) demonstrated a strong degree of agreement between the predicted outcomes and actual values of the model. The clinical decision curves of the predictive model in the training and validation sets were better than the two extreme end lines, as could be observed from the clinical decision curve analysis (**Figures 6**, **7**), suggesting that the model has high clinical practical value. There was a net benefit in clinical application for the training group when predicting postoperative recurrence of usual-type cervical cancer under the effective threshold of >0.19, and the validation group also reported that the model performed well.

### 3.4 Patient information after recurrence

After an additional follow-up of 87 patients with surgical recurrence, the average survival period after recurrence was 15.7 months. Of them, eight patients experienced both internal and exterior pelvic recurrences, 46 patients experienced internal pelvic recurrence, and 33 patients experienced external pelvic recurrence.

## 4 Discussion

Recurrent cervical cancer is a term used to describe the clinical recovery that occurs following radical radiotherapy or standardized initial surgical treatment (radical cervical cancer surgery) and the subsequent recurrence of tumor lesions of the same histological type in the body over time. Depending on the original treatment mode, recurrent cervical cancer can be classified as recurrence after radiotherapy or surgery. Recurrence after surgery indicates the appearance of new tumor lesions after 6 months of surgical treatment, and recurrence after radiotherapy indicates the formation of new tumor lesions after 3 months of intense radiation therapy. Based on the site of recurrence, recurrence is classified as internal or external pelvic recurrence. Internal pelvic recurrence is further classified as central (limited to the uterus and vagina) or noncentral (pelvic lymph nodes and pelvic wall), whereas external pelvic recurrence refers to lymph node or long-term metastasis outside the pelvic cavity (liver, lung, and kidney).According to previous studies, 14–57% of relapses after surgical therapy occur only in the pelvis, whereas 15–61% occur as distant metastases (8). In this study, the recurrence rate was 22.0% (87/395), which was comparable to previous findings.

Cervical cancer recurrence is associated with factors such as tumor biology, nonstandard diagnosis and treatment, and individual variability. Previous studies on cervical cancer recurrence have not distinguished between histological types or used only histological types as independent variables, resulting in a relatively small number of cases of cervical adenocarcinoma. According to Rudtanasudjatum et al. (9), the risk of early cervical cancer recurrence was comparable to that of squamous cell carcinoma and adenocarcinoma. However, Mabuchi et al. (10) demonstrated that adenocarcinoma was an independent risk factor for recurrence. Furthermore, cervical adenocarcinoma responds to treatment more slowly than does squamous cell carcinoma (11), which frequently expands into the deep cervical myometrium, infiltrates the periuterine and lymphatic regions, and may be associated with a higher risk of recurrence. There is still considerable debate in current studies about whether its histology influences recurrence (12, 13).

Cervical squamous cell carcinoma (70%) and cervical adenocarcinoma (25%) are the two most common types of cervical cancer, which are further classified as cervical adenocarcinoma, cervical squamous cell carcinoma, and neuroendocrine carcinoma according to the current international classification. As there are currently numerous types of cervical adenocarcinoma, the object for this study was the most prevalent type with an increasing incidence rate. In this study, we constructed a predictive model for the recurrence of usual-type cervical cancer by excluding characteristics that could influence nonstandard diagnosis and therapy. The independent risk factors for recurrence were tumor size, postoperative HPV infection time, FIGO staging, and peripheral nerve infiltration. The model was validated and confirmed to have a high clinical practical value.

Young women with cervical cancer are more likely to experience a poor prognosis from rare types of cervical adenocarcinoma and neuroendocrine carcinoma, which increases the probability of recurrence. Additionally, young women are more likely to experience tumor recurrence and have quicker rates of cell proliferation. Moreover, HPV preferentially infects the bigger cervical ectropion and transition zone, which may contribute to the high incidence of HPV infection in young women. This is also associated with frequent contact with new sexual partners (14). However, in our study, age was an influencing factor in the univariate analysis, but in the multivariate analysis, there was no significant difference in age between the relapse and non-relapse groups.

Both viral (genotype, viral load, and integration) and host (genetics, immunosuppression, and social behavior) factors can affect the duration of HPV infection. Currently, persistent HPV infection is not well-defined. Several researchers believe that when HPV invades host basal cells, it is called persistent HPV infection if a woman’s cervical HPV test consistently shows positive results for the same type during two consecutive follow-up visits spaced 4–6 months or 6–12 months apart (15). In this study, postoperative HPV infection time was defined as follows: it is calculated from the date of surgery to the time of HPV infection detection, and the first negative conversion occurs following a follow-up examination of the vaginal stump shedding cells. A total of 105 patients were not tested for HPV during the preoperative examination for this study, and 71 results were negative. Following surgery, HPV was re-examined in all patients in this study, and several individuals with negative preoperative HPV test results tested positive for the virus after surgery. Currently, >95% of the usual types of cervical adenocarcinomas are HPV-related adenocarcinomas; therefore, it is possible that the preoperative sample selection was inadequate or that a small percentage of patients developed an infection after surgery.

The initial follow-up period following standard surgery is 3 months from the date of surgery; however, this period may significantly vary depending on the patient for personal reasons. The patient tested negative for HPV during postoperative follow-up, indicating a continuous infection duration of 0. There were three types of patients in this group: those who were infected before surgery and not infected after surgery, owing to the possibility of virus self-clearance by the body’s immune system; those who were not tested before surgery and were not infected after surgery, owing to the possibility of partial non-HPV infection and partial HPV virus clearance; and those who tested negative before surgery and negative after surgery, owing to the possibility of non-HPV infection.

Numerous studies have found minor changes in the HPV model and infection time with respect to the effects of persistent HPV infection time on recurrence. Persistent high-risk HPV (HR-HPV) infection is a risk factor for HPV-associated cervical cancer recurrence. Persistent infection weakens a patient’s immune system, promotes tumor growth, and leads to cervical cancer recurrence (16). According to an increasing number of studies, effective surveillance of HR-HPV infection after the initial standardized therapy is an essential predictor of recurrence. In the present study, we found that persistent postoperative HPV infection for >9 months was an independent risk factor for recurrence (p<0.05). Furthermore, the location of recurrence is associated with HR-HPV infection, and the sustained positive incidence of HR-HPV infection in patients with pelvic recurrence is higher than that in patients with distant recurrence. However, no comparison was made in the present study. A study of 113 patients with cervical cancer (stages I–IV) revealed that chronic HPV-18 infection could predict recurrence (17). However, in a study that involved 248 participants who were followed up for 5 years, HPV status had no effect on the recurrence rate (p=0.384) (18). Belkic et al. found that HPV-18 positivity during follow-up was the greatest predictor of recurrence in a cohort of 84 women with cervical adenocarcinoma in situ, with an odds ratio of 141 (19). HPV-18 positivity is reportedly the best predictor of recurrence (p<0.005). Positive HPV findings in two cases predicted recurrence (p<0.02). HPV-18 and prolonged HPV positivity are highly predictive of recurrence (19).

In this study, we discovered that the preoperative HPV-negative group had a higher recurrence rate than the preoperative HPV-positive group(36.84%, 16.44%), which was statistically significant(p=0.0157). Among the HPV-positive group, the recurrence rate of 18 positives was higher than that of 16 positives (5.48%, 5.02%), but the difference was not significant. The vast majority of usual-type cervical adenocarcinoma is HPV-related, and HPV-negative test results are typically explained by insufficient sampling or poor previous testing techniques. However, HPV-negative patients exhibit distinct characteristics and have a poorer prognosis than HPV-positive patients (20). According to reports, the HPV negative rate varies depending on geographical region, histological subtypes, patient age, and sampling material storage time (21). In large-scale epidemiological studies, the HPV positivity rate in usual-type cervical adenocarcinoma ranges between 72 and 90% (21). In this study, the pre-operative HPV detection rate was 73.4%, while the positive HPV rate was 75.5%. As demonstrated in this study, HPV negative is associated with a poor prognosis, including postoperative recurrence (20, 22). However, in a study of cervical adenocarcinoma, there was no significant difference in cancer-specific survival rates between HPV-positive and negative cases (23). However, in other HPV-related malignant tumors, such as head and neck cancer (24), HPV positivity is also associated with a favorable prognosis. This is due to differences between the groups, as HPV-positive tumors are thought to be more susceptible to radiation.

The three main surgical methods for radical hysterectomy in cervical cancer are minimally invasive, open, and robotic. The third edition NCCN guidelines updated in 2019 indicate that laparoscopic surgery for cervical cancer be avoided. The foundation is based on the Anderson Cancer Center’s Laparoscopic Approach to Cervical Carcinoma study (25), which found that for early cervical cancer, minimally invasive surgery may have a greater postoperative recurrence risk than that by open surgery. A meta-analysis revealed no significant difference in the long-term recurrence rate between laparoscopic and robot-assisted laparoscopic surgeries (26); however, there were frequent differences in blood loss and exhaust time during surgery. In a study of 319 patients with cervical cancer who were randomly assigned to either a minimally invasive surgery or laparotomy group, the rates of postoperative adjuvant treatment were comparable between the two groups. A reduced postoperative recurrence rate was associated with less invasive radical hysterectomies (27). However, despite significant differences in univariate analysis, multivariate analysis revealed that the different surgical methods did not significantly influence recurrence (28). There was no significant difference in the postoperative recurrence of usual-type cervical cancer between open and laparoscopic surgeries in this study, which could be due to the small sample size. The use of uterine lifting devices, pneumoperitoneum, vaginal disconnection, and suturing may be associated with an increased risk of postoperative recurrence during laparoscopic surgery.

The role of clinicopathological factors in the postoperative recurrence of cervical cancer remains controversial. In the present study, the factors that influenced postoperative recurrence were FIGO stage, tumor size, ovarian metastasis, lymph node metastasis, vascular infiltration, depth of the infiltrating muscle layer, and peripheral nerve invasion (p<0.05). FIGO stage and peripheral nerve invasion were independent risk factors for postoperative recurrence in usual-type cervical cancer in multivariate analysis. Compared with the 2009 FIGO staging system, the 2018 FIGO staging system has switched to a pathological staging approach for cervical cancer surgery.

The spread of tumor cells through lymphatic veins or blood causes postoperative recurrence (29). LVSI is more common in patients with recurrent cervical cancer, which may be due to LVSI generating distant hematogenous metastases (30). According to a previous study, the incidence rate of lymph node metastasis in LVSI-positive patients was higher than that in LVSI-negative patients (31). LVSI did not have a significant effect on recurrence in the multifactor analysis in this study, and this may be associated with LVSI-positive patients receiving more adjuvant treatment after surgery, which is comparable to the findings of the study by Wey (32). Perineural infiltration is defined as tumor invasion of neural tissues. This peripheral nerve infiltration also confirms the spread of malignant cells.

The lymph node status is associated with cervical cancer recurrence. Mabuchi et al. (33) analyzed 163 cases of cervical adenocarcinoma and adenosquamous cell carcinoma in FIGO 2009 stages IA2–IIB and concluded that lymph node metastasis was a significant predictive factor for cervical adenocarcinoma and adenosquamous cell carcinoma. Patients with lymph node metastases show a dramatically decreased disease-free survival. Meir et al. reported that lymph node metastasis was an independent risk factor for recurrence. The most recent 2018 FIGO staging system defines lymph node metastasis as stage IIIC, confirming that lymph node metastasis may result in a worse prognosis. This was confirmed by the results of the present study. The prognosis differs slightly in patients whose cervical cancer lymph nodes have metastasized. Pelvic lymph nodes, including the internal and external iliac lymph nodes, are not independently associated with poor prognosis in individuals with recurrence, whereas iliac lymph node metastases are. Consequently, further studies with large sample sizes are required to assess the prognostic variations and their effects on recurrence among individuals who have positive iliac common lymph nodes and positive iliac internal and external lymph nodes.

According to the latest guidelines, ovarian preservation is not an absolute contraindication for usual-type cervical adenocarcinoma, and the indications for ovarian preservation are still debated. Ovarian metastasis is considered a risk factor for cervical adenocarcinoma metastasis. Therefore, ovarian preservation is not recommended for patients with adenocarcinoma. However, in this study, preservation of the ovary and ovarian metastasis were not risk factors for postoperative recurrence of usual-type cervical adenocarcinoma in the multifactor analysis. Considering that cervical adenocarcinoma and ovaries are both glandular tissues, it is still necessary to be cautious in grasping the indications for ovarian preservation.

Currently, there are disparities in the recurrence rates of cervical cancer after surgery among different treatment approaches. Chemotherapy is a systemic treatment that can reduce distant recurrence; however, investigations have shown that this is not the case. In one trial, chemotherapy reduced local recurrence rates but had no effect on distant recurrence, which could be due to the confounding effects of adjuvant therapy such as postoperative radiation therapy (34). In a trial of 246 patients who required further postoperative chemotherapy, 182 received it, with a postoperative recurrence rate of 2.74%, whereas 64 did not, with a recurrence rate of 10.93% (p<0.05) (34). In the present study, adjuvant treatment had a considerable effect on postoperative recurrence. Therefore, more stratified, large-sample testing is required. According to Rotman et al. (35), pelvic radiation therapy after radical surgery can considerably reduce the incidence of recurrence and progression-free survival in women with stage Ib cervical cancer. Sakai et al. (36) divided 122 patients with early cervical cancer who underwent thorough hysterectomy into four groups: paclitaxel+cisplatin adjuvant chemotherapy (n = 82), other chemotherapy (n = 10), radiotherapy (n = 25), and no further treatment (n = 5). The results showed that there was no difference in the overall 5-year survival rate of the abovementioned patients (p>0.05); however, when subgroup analysis was performed only for patients with high-risk factors, recurrence-free survival (RFS) time was significantly shorter in the radiotherapy group than in the paclitaxel+cisplatin adjuvant chemotherapy group. This suggests that for patients with cervical cancer with high-risk factors, chemotherapeutic medications can improve radiation sensitivity and minimize the probability of postoperative recurrence. Takekuma et al. (37) randomly assigned 111 postoperative patients with stage IB–IIB cervical cancer and high-risk variables to one of the two groups: chemotherapy (n = 37) or synchronous radiochemotherapy (n = 74). The results showed that the chemotherapy and radiochemotherapy groups had 4-year RFS rates of 71.7% and 68.3%, respectively (p>0.05). According to this study, the efficacies of synchronous radiotherapy and c.

Currently, there are different adjuvant treatment methods for different stages, preoperative and postoperative stages of cervical cancer, mainly including neoadjuvant treatment for patients with stage Ib3 and above disease or radical radiotherapy for patients with disease in the later stages. The effectiveness of various adjuvant treatment approaches in patients with advanced cervical cancer is currently under debate. In a study that included patients with advanced adenocarcinoma or adenosquamous cell carcinoma, the 5-year overall survival and RFS rates of the radical hysterectomy (n = 128) and synchronous radiotherapy and chemotherapy (n = 36) groups were 83.2% and 73.3% (p=0.164) and 75.2% and 59.6% (p<0.036), respectively. Patients who underwent radical hysterectomy had a lower probability of recurrence (11.6%, p=0.023) (38). There is an ongoing debate regarding whether neoadjuvant therapy can improve patient survival and minimize recurrence rates. Some clinical trials have demonstrated that neoadjuvant therapy can further reduce tumor volume and improve surgical treatment effectiveness and prognosis. Chen et al. (39) found that compared with patients who underwent surgery alone, patients who underwent neoadjuvant chemotherapy-assisted surgery had significantly better 3- and 5-year survival rates (p<0.05). According to a systematic review, although neoadjuvant chemotherapy reduces postoperative recurrence, there is no evidence that it influences the survival rate of patients with cervical cancer at various stages and periods (40).

This study has the following limitations: (1) It was a retrospective study; therefore, inherent biases, such as those regarding data inclusion, are possible. (2) The case data were obtained from the same institution, and the treatment techniques and environment were uniform, indicating a lack of external validation. (3) Although pathological analysis is the gold standard for detecting recurrence, some individuals have advanced illnesses that can only be detected through imaging. Currently, there are differences in the recurrence rates of cervical cancer after surgery across different treatment modalities.

## 5 Conclusions

The model and nomogram for predicting the recurrence of usual-type cervical adenocarcinoma after surgery are accurate and effective, with high discrimination and calibration, and have good clinical practical value, based on FIGO staging, peripheral nerve invasion, tumor size, and postoperative HPV infection months. However, a thorough assessment of postoperative recurrence is critical, and large-scale stratified assessments of the risk of cervical cancer recurrence are required in the future. In addition, further studies on the management of various types of recurrence in common cervical adenocarcinomas are required.

## Data availability statement

The raw data supporting the conclusions of this article will be made available by the authors, without undue reservation.

## Ethics statement

The studies involving humans were approved by Ethics Committee of Shengjing Hospital Affiliated to China Medical University. The studies were conducted in accordance with the local legislation and institutional requirements. Written informed consent for participation in this study was provided by the participants’ legal guardians/next of kin. Written informed consent was obtained from the individual(s) for the publication of any potentially identifiable images or data included in this article.

## Author contributions

YL: Data curation, Formal analysis, Investigation, Methodology, Validation, Writing – original draft. NZ: Writing – review & editing, Data curation, Resources. QY: Formal analysis, Funding acquisition, Project administration, Supervision, Writing – review & editing.
